# Neurochemical and Energetic Alterations in Depression: A Narrative Review of Potential PET Biomarkers

**DOI:** 10.3390/ijms27031267

**Published:** 2026-01-27

**Authors:** Santiago Jose Cornejo Schmiedl, Bryan Astudillo Ortega, Bernardo Sosa-Moscoso, Gabriela González de Armas, Jose Ignacio Montenegro Galarza, Jose A. Rodas, Jose E. Leon-Rojas

**Affiliations:** 1 NeurALL Research Group, Quito 170157, Ecuador; santicornejo123@gmail.com (S.J.C.S.); bryan_astu@hotmail.es (B.A.O.); bsosa413@gmail.com (B.S.-M.); 2 Escuela de Medicina, Universidad de Las Américas, Quito 170124, Ecuador; gabriela.gonzalez.dearmas@udla.edu.ec (G.G.d.A.); jose.montenegro.galarza@udla.edu.ec (J.I.M.G.); 3 School of Psychology, University College Dublin, D04 V1W8 Dublin, Ireland; josea.rodasp@gmail.com; 4 Escuela de Psicología, Universidad Espíritu Santo, Samborondón 092301, Ecuador; 5 Grupo de Investigación Bienestar, Salud y Sociedad, Escuela de Psicología y Educación, Universidad de Las Américas, Quito 170124, Ecuador

**Keywords:** depression, positron emission tomography, PET, biomarker, brain metabolism

## Abstract

Depression is a heterogeneous neuropsychiatric disorder with variable clinical presentation and response to treatment. This variability has motivated interest in neuroimaging biomarkers capable of disease characterization and therapeutic prediction. Positron emission tomography (PET) enables in vivo assessment of cerebral glucose utilization, neurochemical targets, inflammatory markers, and cerebral blood flow. This narrative review synthesizes PET studies conducted predominantly in adults with major depressive disorder diagnosed using DSM-based criteria, with bipolar disorder included only when imaging was performed during a depressive episode. Studies were identified through a structured, non-systematic literature search of major databases. Depression is consistently associated with regionally specific PET alterations within cortico-limbic and cortico-striatal circuits; studies most frequently report reduced glucose-derived PET measures in prefrontal and anterior cingulate regions at baseline, with treatment responders showing relative increases or redistribution of these measures following interventions. Neurochemical PET studies demonstrate altered receptor, transporter, or enzyme-related binding in serotonergic, dopaminergic, and noradrenergic systems, while neuroinflammatory and perfusion studies reveal regionally increased PET signals in subsets of patients. Overall, PET findings indicate convergent, region-specific and neurochemical alterations associated with depressive episodes and treatment response. Interpretation is constrained by methodological and clinical heterogeneity, underscoring the need for harmonized, longitudinal PET studies.

## 1. Introduction

Depression is a common and heterogeneous neuropsychiatric disorder with substantial global burden and highly variable clinical trajectories. Despite the availability of pharmacological, psychotherapeutic, and neuromodulatory interventions, a sizeable proportion of patients (around 40%) do not respond adequately to first-line treatment, and many experience delays in achieving symptom remission due to empirical trial-and-error medication selection [1]. This therapeutic uncertainty has intensified interest in objective neurobiological biomarkers that can support disease characterization and, crucially, predict treatment response [2]. From a neurobiological perspective, depression is increasingly conceptualized as a disorder of distributed cortico-subcortical network dysfunction rather than a purely psychological or monoaminergic condition [1,2]. Converging evidence implicates prefrontal and cingulate regions involved in cognitive control and affecting regulation, alongside subcortical structures such as the basal ganglia, thalamus, hippocampus, amygdala, and midbrain nuclei that support reward processing, motivation, stress responsivity, and emotional salience [3,4,5,6,7,8,9,10,11,12,13,14,15]. These networks are modulated by energetic metabolism, monoaminergic neurotransmission, neuroimmune signaling, and neurovascular coupling, all of which are processes that can be interrogated in vivo using nuclear imaging. Framing depression in this systems-level context provides a clear biological rationale for molecular neuroimaging approaches and supports interpretation of nuclear-derived alterations as network-level correlates of depressive episodes rather than isolated regional abnormalities.

Certainly, neuroimaging has contributed to this effort by enabling in vivo analysis of brain networks implicated in mood regulation, cognitive control, and stress processing [3,4,5,6,7,8,9,10,11,12,13,14,15]. Among available modalities, positron emission tomography (PET) offers distinct strengths because it permits quantitative assessment of regional cerebral metabolism, neurotransmitter related targets, neuroinflammatory signals, and cerebral perfusion using radiotracers tailored to specific biological processes [3,4,5,6,7,8,9,10,11,12,13,14,15]. By mapping functional alterations in vivo, PET complements structural approaches and can identify state-dependent changes associated with depressive episodes and treatment effects [3,4].

Across PET studies, depression has been associated with reproducible abnormalities within interconnected cortico-limbic and cortico-striatal circuits; frequently implicated regions include the prefrontal cortex, anterior cingulate cortex, orbitofrontal cortex, hippocampus, amygdala, basal ganglia, and thalamus, all of which contribute to emotional salience, motivation, reward learning, and executive regulation [2,5,10,11,12]. At the energetic level, 18F-fluorodeoxyglucose PET studies commonly report altered regional glucose alterations, often involving reduced uptake in prefrontal and cingulate areas, alongside treatment-related metabolic shifts that differ between responders and non-responders [5,7,8,9,10]. At the neurochemical level, PET studies employing serotonergic, dopaminergic, and noradrenergic radioligands reveal disturbances in monoaminergic systems across both cortical and subcortical structures, with patterns that vary according to clinical phenotype, treatment modality, and outcome [1,6]. Additional PET evidence implicates monoamine oxidase activity and disruptions in cerebral blood flow as complementary components of depressive pathophysiology [3,13]. Importantly, these PET-derived abnormalities can be interpreted within contemporary models that conceptualize depression as a disorder of network dysregulation rather than a single neurotransmitter deficit; monoaminergic alterations may influence synaptic efficiency and neurovascular coupling, glucose metabolism reflects the energetic demands of altered neuronal activity, and perfusion changes mirror dynamic modulation of these circuits in response to treatment [2,5,8,14]. From a clinical perspective, PET therefore represents a promising tool to identify convergent biological signatures of depression and to support stratification of patients according to likely treatment response, particularly when baseline PET patterns differentiate responders from non-responders across interventions [2,5,14,15].

In this narrative review, we synthesize the current PET literature on neurochemical and energetic alterations in depression, with emphasis on (i) regional glucose alterations, (ii) serotonergic, dopaminergic, and noradrenergic systems, (iii) monoamine oxidase related measures, and (iv) inflammation and cerebral perfusion. We further integrate these findings in relation to established cortico-limbic circuitry and discuss their potential value as biomarkers for disease characterization and treatment prediction, while acknowledging current methodological heterogeneity and the need for standardized longitudinal designs. Accordingly, our narrative review aims to provide an integrative overview of PET-derived metabolic alterations in depression across multiple biological domains rather than focusing on a single mechanistic pathway; our objective is to synthesize convergent patterns across these systems and highlight their potential interconnections within established neurobiological models of depression to provide an introductory framework for readers interested in understanding more about the potential role of PET in depression.

## 2. Methodology

The included studies encompassed heterogeneous clinical populations, including patients with treatment resistant depression, late-life depression, recent suicidal behavior, and individuals undergoing a depressive episode within bipolar disorder. These clinical characteristics were not used as exclusion criteria but rather were retained to reflect the real-world heterogeneity of depressive disorders and to contextualize PET findings across different clinical phenotypes. PET studies employing non-FDG radiotracers were included when they assessed biological processes with a direct or indirect relationship to cerebral metabolism, including monoaminergic neurotransmission, monoamine oxidase activity, neuroinflammation, and cerebral blood flow. Although these tracers do not measure glucose uptake per se, they capture molecular and physiological processes that modulate synaptic activity, neurovascular coupling, and energetic demand within cortico-limbic circuits. Consequently, their inclusion was considered relevant to the review objective of characterizing metabolic and neurochemical alterations associated with depression and treatment response. We also included articles in both English and Spanish without any temporal restrictions. We excluded other literature reviews, conference abstracts, editorials, and commentaries.

PubMed, Scopus and the Virtual Health Library (VHL) were queried from inception until August 2025 without filters. The search combined terms related to depression and PET imaging, including the following: “major depressive disorder”, “depression”, “bipolar depression”, “treatment resistant depression”, “PET”, “positron emission tomography”, “FDG”, “glucose metabolism”, “cerebral blood flow”, “serotonin”, “dopamine”, “norepinephrine”, “monoamine oxidase”, “TSPO”, and “neuroinflammation”. Reference lists of eligible articles and relevant reviews were also screened to identify additional studies.

Eligible studies included participants with unipolar major depressive disorder diagnosed using DSM IV (given that it was the most commonly used diagnostic framework during the time of publication of the articles) or any DSM-based criteria (relevant to the year of publication) and, when relevant, participants with bipolar disorder only if imaging was explicitly performed during a depressive episode (bipolar depression). Because bipolar depression is clinically distinct from unipolar depression, we retained diagnostic category as an a priori synthesis variable and reported findings separately where sufficient information was available.

Data were extracted using a predefined template in Microsoft Excel capturing study design; sample size; diagnostic framework and depression subtype; age group; medication status at scanning (medication free versus medicated) when reported; treatment resistance status when reported; tracer and biological target; acquisition and quantification approach; regions of interest and analytic strategy; primary baseline findings; and, when applicable, treatment modality and clinical outcome definition (for example, response or remission). Owing to heterogeneity in tracers, analytic pipelines, clinical phenotyping, and outcome definitions, a quantitative meta-analysis was not pursued. Instead, we conducted a structured narrative synthesis organized by imaging domain and tracer target, while explicitly highlighting the influence of major clinical modifiers.

Across included longitudinal treatment studies, “response” and “remission” were defined according to the original authors’ criteria, which most commonly relied on validated symptom scales such as the Hamilton Depression Rating Scale (HDRS/HAM-D), Montgomery–Asberg Depression Rating Scale (MADRS), or comparable clinician-rated instruments, assessed at modality-specific follow-up timepoints. When explicitly reported, response was typically operationalized as a prespecified percentage reduction from baseline severity (for example, ≥50% reduction), whereas remission was defined by falling below a scale-specific threshold. Because thresholds, scales, and follow-up durations varied between studies and treatment modalities (pharmacotherapy, psychotherapy, and neuromodulation), we did not impose a single harmonized cut-off. Instead, we extracted each study’s response definition (scale, threshold, and timepoint) and interpreted results within that context. For synthesis, studies were categorized a priori into three comparison types: (1) baseline predictors of later treatment response, where pre-treatment PET measures were compared between participants who later met response/remission criteria versus those who did not; (2) pre–post treatment changes, where PET measures were compared within participants before and after an intervention; and (3) cross-sectional differences, including patient–control contrasts or subgroup comparisons without longitudinal outcome assessment. This classification was used to avoid conflating predictive biomarkers with state-associated findings.

To reduce interpretive ambiguity, findings were synthesized with explicit attention to key sources of heterogeneity that commonly affect PET outcomes. Where reported, results were contextualized according to medication status, treatment resistance status, age group including late-life cohorts, diagnostic category (unipolar versus bipolar depression), and therapeutic modality (pharmacotherapy, psychotherapy, and neuromodulation). When subgroup details were not provided in the primary study, this was recorded as a limitation and interpreted cautiously. Finally, for the purposes of this review, the term “metabolic alterations” is used in a broad neurobiological sense, encompassing energetic metabolism, neurotransmitter turnover, enzymatic degradation, inflammatory activity, and perfusion processes that collectively shape cerebral metabolic function.

## 3. PET Findings

PET studies included in this review reported a range of quantitative outcome measures depending on tracer, acquisition protocol, and analytic approach. For FDG PET, reported metrics included absolute cerebral metabolic rate of glucose (CMRglc) and relative measures such as standardized uptake values or normalized regional uptake ratios. These measures are not interchangeable and were interpreted within the context of each individual study. For clarity, we refer to findings using the specific metric reported by the original authors when available or describe relative regional differences without implying absolute metabolic quantification when metrics were not explicitly stated.

### 3.1. PET Alterations

#### 3.1.1. Glucose PET Alterations

In PET/CT imaging, it is possible to assess alterations in glucose with a [18F] fluorodeoxyglucose radioactive tracer. FDG PET studies quantify regional glucose utilization using absolute or relative uptake measures, which serve as proxies for underlying neuronal and glial metabolic demand [3,4]. Accordingly, we identified 20 studies that assessed regional glucose utilization using FDG PET in depressed patients across different regions of interest, employing either absolute or relative measures, with and without pharmacological treatment [3,4,5,6,7,8,9,10,11,12,13,14,15,16,17,18,19,20,21,22].

There are several non-pharmacological treatments that used 18F-PET/CT as a biomarker. For example, in responders to transcranial magnetic stimulation, FDG PET demonstrated higher relative measures in several ROIs, including the left hemisphere of the prefrontal cortex (PFC), orbitofrontal cortex (OFC), subgenual cingulate, anterior insula, and the temporal lobe as well as the parahippocampus and amygdala [3]. Another study reported relatively increased FDG uptake in the anterior cingulate cortex (ACC) as well [4]. Patients treated with deep-brain stimulation (DBS) demonstrated increased prefrontal FDG uptake following treatment.

Regarding pharmacologic treatment, some studies showed that non-responders to standard-dose venlafaxine exhibited higher baseline FDG uptake in the region spanning the pregenual and subgenual cingulate cortices compared with responders [5]. Furthermore, patients who responded well to sertraline showed an increased FDG uptake in the left lateral orbital cortex, the left amygdala, and the posterior cingulate cortex (PCC) [6], while paroxetine responders showed increased activity in the dorsolateral and ventrolateral parts of the prefrontal and parietal cortex and dorsal anterior cingulate, while also inducing a decrease FDG uptake in the subgenual cingulate and in the anterior and posterior insular regions, right hippocampal, and parahipocampal regions [7]. Patients who responded well to both standard-dose venlafaxine and cognitive behavioral therapy (CBT) showed a similar finding as treatment alone, meaning an increase in FDG uptake in the right occipital–temporal cortex while inducing a decrease in the orbitofrontal and left medial prefrontal cortex [8], while patients who responded to CBT and paroxetine showed an increase in the left insula and temporal lobe [9]. Other studies have postulated that some regions show a decrease in FDG uptake, such as the ACC and the anterolateral/medial dorsal prefrontal cortex in patients with untreated depression [10], with lower glucose uptake rates found in the PFC, frontal cortex, the superior frontal gyrus, the middle frontal gyrus, the inferior frontal gyrus, the inferior parietal lobe, and the superior parietal lobe [11]. Additionally, activation has been shown to be diminished in depressive patients in the ACC, striatum, caudate, dlPFC, and rostral PFC [12]. Finally, another study found decreased FDG uptake in the PFC, orbitofrontal area, left dorsolateral prefrontal cortex (dlPFC), the temporal lobes, and left ACC in patients responding to repeated TMS [13]. Conversely, responders to selective serotonin reuptake inhibitors (SSRIs) had a reduction in midbrain, right parahippocampal gyrus, right putamen, right lateral globus pallidus, ventral limbic structures, and right thalamus [14]. More specifically, responders to sertraline showed a reduction in the right inferior frontal gyrus and right cerebellum [15], as well as the ACC and medial and dlPFC [10], whereas responders to paroxetine showed a decrease in the caudate and putamen [7] and a decrease in the antero-inferior temporal lobe when associated with CBT [9].

Studies that assessed glucose during the course of treatment found that patients treated with citalopram showed an increase uptake in the right putamen, left right occipital cortex, and the left cerebellum, while the uptake rate was decreased with the same drug in the left-middle-frontal cortex and the left inferior parietal lobule [16]. Patients with dual therapy, consisting of SSRIs and vagal nerve stimulation (VNS), showed further decrease in the left medial frontal gyrus, left area subcentralis, left inferior and right medial temporal gyrus, left caudate head, limbic system, and brainstem [17]. On the other hand, patients with bipolar depressive disorder showed decreases in FDG uptake in the lateral frontal region, lateral temporal and medial temporal cortices, the basal ganglia, and the thalamus when compared to controls during treatment with benzodiazepines and/or lithium [18].

Specific post-treatment changes have also been documented in PET/CT studies in patients with depression. Patients that responded to TMS showed decreased activity (i.e., decreased glucose metabolic rate) in the right medial temporal lobe [19]. A decline in the insular glucose in responders to psychodynamic psychotherapy predicted a decline in scoring of depression scores [20]. Clonidine showed a decrease in FDG-derived metabolic measures in the cerebellum and thalamus [21], while standard-dose venlafaxine responders showed a decrease in the right posterior temporal and bilateral superior temporal gyri [22].

Taken together, FDG PET studies suggest that depression is characterized by altered glucose utilization and uptake within prefrontal and cingulate regions, with treatment-related changes differing by therapeutic modality. Neuromodulatory interventions such as transcranial magnetic stimulation and deep brain stimulation are generally associated with increased or redistributed prefrontal and cingulate FDG uptake in responders, whereas pharmacological treatments show more heterogeneous patterns, often distinguishing responders from non-responders by baseline regional PET alterations. Psychotherapeutic interventions are likewise associated with region-specific modulation rather than global normalization. These patterns support the interpretation of glucose as a state-sensitive marker linked to treatment response rather than a uniform trait abnormality.

#### 3.1.2. Serotonin PET Alterations

Serotonin is one of the key neurotransmitters involved in the pathogenesis of depression. As such, it stands to reason that serotonin alterations will be frequent in depressed individuals. Patients with major depressive disorder have shown a decrease in serotonin uptake in PET, reflecting a decrease in receptor or transporter binding in the midbrain, thalamus, striatum, amygdala, anterior cingulate cortex, dorsal raphe nuclei, dlPFC, inferior, medial and superior temporal gyri, posterior cingulate cortex, and right premotor area [23]. Furthermore, suicidal ideations have also been related with lower serotonergic uptake, particularly in the midbrain and thalamic areas [11]. Another study showed that treatment-resistant patients showed lower serotonin receptor binding capacity in the overall cortex, especially in key projection sites such as the prefrontal regions and the raphe nuclei, related to higher lethality and suicide attempts, as well as more severe ideations [24]; patients that are resistant to treatment showed lower serotonin binding in the frontal and dorsolateral prefrontal cortices, as well as lower levels of serotonin release [1].

In patients treated with TMS, there was a decrease in serotonin uptake in the right hippocampus after stimulation and a reduction in the right dlPFC [25]. Non-responders showed an increase in serotonin uptake in the left amygdala, uncinate fasciculus, and anterior commissure but lower in the left orbital cortex [26]. Patients undergoing SSRI therapy showed an increase in serotonin binding potential in the dorsal anterior cingulate cortex, subgenual and pre-subgenual anterior cingulate cortex, and medial orbital cortex [27], while non-responders showed an increase in the same index in the prefrontal cortex, midbrain, thalamus, striatum, and medial orbital cortex [28]. Likewise, a response to SSRIs was associated with lower global serotonin availability [29], which could be interpreted as an increase in serotonin use in key areas. Citalopram and escitalopram users showed an increased uptake in the amygdala–anterior hippocampus complex, in the habenula, putamen, orbitofrontal cortex, the subgenual and anterior cingulate cortex, insula, midbrain, hippocampus, and globus pallidum [30]. Paroxetine users had a decreased serotoninergic uptake in the medial frontal gyrus, lateral orbitofrontal cortex, parahippocampal gyrus, posteromedial temporal gyrus, basal ganglia, brainstem, rostral anterior cingulate, and ventrolateral prefrontal cortex [31].

#### 3.1.3. Dopamine PET Alterations

Dopaminergic PET studies reported altered dopamine transporter or receptor binding in depressed patients, with a lower metabolic rate evidenced in the bilateral putamen, nucleus accumbens, and ventral tegmental area, more evidently in depressed patients with psychomotor retardation [32]. Furthermore, a reduction in dopamine transporter availability was related to increasing numbers of depressive episodes; especially in the ventral tegmental area, availability was lowest in individuals who reported feeling trapped in stressful circumstances [33]. After receiving treatment with either standard-dose fluoxetine, paroxetine or venlafaxine, the striatal/occipital binding ratio of the ligand was higher in patients with major depression, which could translate to a higher availability of dopamine receptors in the striatal cortex of depressed patients [34]. In another study that evaluated response to treatment, Dopamine-D2 receptor binding of the radiotracer increased in the anterior cingulate gyrus and in adjacent parts of the frontal lobe as well as in the striatum in patients treated with SSRI and/or benzodiazepines [35]; on the other hand, binding decreased in the anterior cingulate gyrus and in adjacent parts of the frontal lobe as well as in the striatum in non-responders [35]. Finally, patients treated with bupropion showed an increase in receptor occupancy; however, this was not related to the clinical response to treatment [36].

#### 3.1.4. Norepinephrine PET Alterations

Few studies analyze changes in norepinephrine transporter binding or occupancy in depressed patients. Concretely, there was a reported decrease in the norepinephrine binding rate in depressed patients, specifically in the thalamus and in the thalamic subregion next to the prefrontal cortex [37]. When subjected to treatment with venlafaxine, a SNRI, the norepinephrine transporter occupancy showed a dose-dependent increase, up to 150 mg/day [38]; on the other hand, higher doses showed a decrease in this receptor’s occupancy in comparison to MDD patients not treated with venlafaxine [38]. Furthermore, another study showed a similar increase in transporter occupancy with the use of nortriptyline, although the dose was not specified [39].

#### 3.1.5. Monoamine Oxidase (MAO) PET Alterations

The enzyme monoamine oxidase (MAO) plays a crucial role in the pathophysiology of depression and is a key target for antidepressant therapy [40]. As such, the study of this enzyme using PET-related techniques can bring further insight into the mechanisms of depression. As such, patients with depression have shown an increased MAO uptake in the prefrontal cortex, anterior cingulate cortex, posterior cingulate cortex, caudate, putamen, thalamus, anterior temporal cortex, midbrain, and the hippocampus and parahippocampus [41]. Furthermore, depressed patients showed an increase in the density of MAO receptor occupancy in the dorsolateral prefrontal cortex, ventrolateral prefrontal cortex, thalamus, and orbitofrontal cortex [42]. On the other hand, there was an increase in the MAO uptake of the prefrontal and anterior cingulate cortices [40]. Elevated MAO binding has been interpreted as consistent with increased monoamine oxidative capacity, although PET cannot directly assess neurotransmitter depletion.

**Figure 1:**
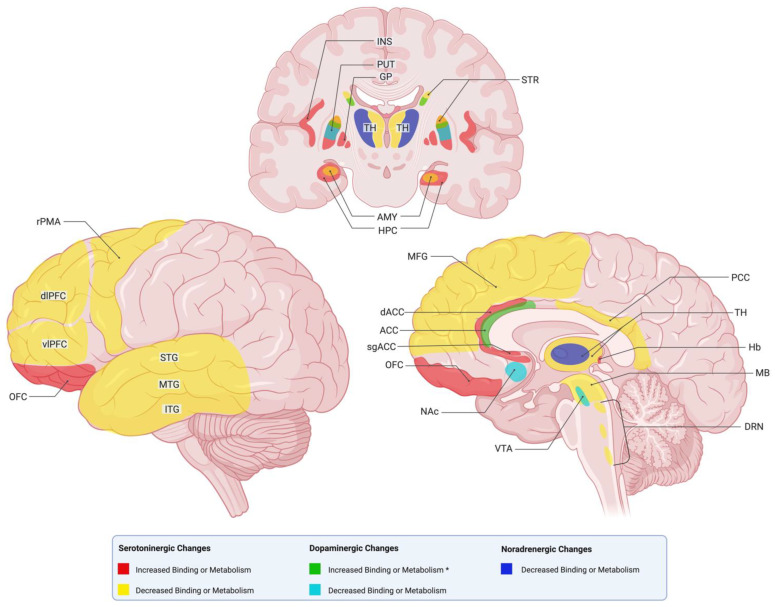
Summary of the brain regions exhibiting increased or decreased monoaminergic PET alterations identified in depression, organized by serotonin, dopamine, and norepinephrine systems. This Figure provides a schematic overview of regional patterns identified across PET domains and is intended as a qualitative synthesis; increased or decreased labeling reflect relative changes in the reported PET metrics and should not be interpreted as direct measures of neurotransmitter concentrations or absolute metabolic rates. * denotes change after treatment only. Created in BioRender. Américas, U. (2026) https://BioRender.com/kv9vih4.

With regard to the effect of antidepressant treatment, the use of moclobemide, a MAO inhibitor, showed a reduction in MAO uptake across all brain regions [43], while a study investigating the use of electroconvulsive therapy showed no changes in the distribution volume of the enzyme [44]. A summary of the main PET alterations can be found in Figure 1.

#### 3.1.6. Other PET Alterations

Opioid alterations have also been evidenced; notably, a decrease in kappa opioid receptor density in the right anterior cingulate, right superior frontal gyrus, and right insula [45]. Additionally, the binding rate of the kappa receptor was related with worse symptom severity and HDRS scoring [46]. Another study showed a lower [11C]-doxepin (histamine receptor) binding in frontal, prefrontal cortices, and cingulate gyrus in depressed patients [47].

Finally, Table 1 showcases a summary of relevant changes in PET tracers according to modality of treatment.

### 3.2. Inflammation State Findings and Cerebral Blood Flow

PET can also be a useful study for inflammatory state analyses, as positron emission and inflammation have a direct correlation. In depressed patients, notably in patients with higher occurrences of suicidal ideations, an increase in inflammation in the anterior cingulate cortex and insula has been previously reported and detected with PET [48]. However, there is a scarcity of these studies which could be a potential future avenue for research.

When looking at cerebral blood flow (CBF), PET is considered as a gold-standard tool, using radioactive tracers such as ^15^O-labeled water [49,50,51,52,53]. For example, a study that evaluated pain tolerance in depressive patients indicated an increase in CBF in the right temporal gyrus, left amygdala, right anterior cingulate cortex, bilateral medial frontal gyrus, bilateral insula, lingual gyrus, right precentral gyrus, and left postcentral gyrus [49]. There were also negative correlations between rCBF and d-HDRS scores in the left parahippocampus region, the thalamus, the left anterior cingulate cortex, and the right precentral cortex [50]. The CBF in pain tolerance-stimulated patients has been shown to be increased in the left middle frontal gyrus [51], while patients undergoing TMS treatment showed an increase in CBF in the prefrontal, parietal, occipital cortex, and anterior/posterior cingulate [52]. This study showed further differences depending on the frequency of stimulation; the 15 Hz group showed increased relative rCBF in the inferior frontal cortices bilaterally, the right dorsomedial frontal cortex, right posterior cingulate, and right parahippocampus and decreased relative rCBF in the right orbital cortex, left uncus, and right subcallosal gyrus [53]. Meanwhile, in the 1 Hz group, significant areas of increased CBF were found in the right dorsal anterior cingulate, left insula, left somatosensory cortex, right parietal cortex (precuneus), left cerebellum (vermis, intermediate zone), and right insula [53]. Finally, another study indicated a relation between the CBF in the right middle temporal cortex, left periinsular, middle temporal cortex, inferior parietal cortex, left inferior frontal cortex, and the midline of the orbitofrontal cortex and the outcomes of electroconvulsive therapy, pointing towards a potential predictive utility of the technique [50].

### 3.3. Other Notable Findings

A study that investigated microglial activity with PET found that in patients with untreated depressive disorder for longer than 10 years, such activity was increased in the anterior and posterior cingulate cortex and the subgenual prefrontal cortex; microglial activity was measured using the translocator protein density, determined by distribution volume (TSPO VT) [54]. Furthermore, greater microglial activity in the prefrontal cortex, anterior cingulate cortex, and insula was found in these patients compared to patients with untreated depression for 9 year or less [54]. Finally, a single study investigated alterations in blood–brain barrier function, where a higher molecular efflux pump P-glycoprotein (P-gp) density potentially correlated with treatment-resistant depression [55].

## 4. Discussion

The advent of imaging studies has brought a proliferation of diverse radiological methods available for research. Likewise, more research is seeking to better characterize psychiatric conditions and to provide potential diagnostic tools (and biomarkers) for everyday psychiatric practice. Imaging studies that use positron-emission tomography (PET) are an example of such a practice. In the current literature, there are various reported alterations in different metabolic pathways that are unique to patients with mood disorders, such as depression. Therefore, our article seeks to summarize said findings and provide an easy reference for individuals looking to delve deeper into the field of nuclear imaging as a potential biomarker for depression.

One of the accepted pathophysiologic characteristics of depression is the disruption of brain neurotransmitters, such as serotonin, dopamine, and norepinephrine [56]. A myriad of pharmacological options were therefore developed for its treatment, such as selective serotonin reuptake inhibitors (SSRI), dual serotonin-noradrenaline reuptake inhibitors (SNRI), monoamine oxidase inhibitors (MAOI), and other antidepressants such as bupropion and mirtazapine, which act over these neurotransmitters and their metabolism [57]. This monoaminergic transmission theory of depression has been the mainstay for over 70 years, and the findings in various PET studies support this theory [57,58]. Concretely, the studies describe alterations in serotoninergic, dopaminergic, and noradrenergic function in depressed patients compared to healthy controls. Serotoninergic metabolic rates, as well as noradrenaline and dopamine binding to the respective radiotracers, have been shown to be reduced [23,32,33,37].

These findings were shown in key brain regions involved in relevant features of depression; for example, the monoamine pathways were affected in the anterior cingulate cortex and areas of the prefrontal cortex, hippocampus, and thalamus. Conversely, the anterior cingulate cortex, an area related to attention and sensory processes, has been shown to be closely involved with emotional circuits and interconnected with other structures such as the amygdala [2,59], acting as a biomarker for predicting response to treatment [2]. The prefrontal cortex is another key region, presenting functional, structural, and connectivity alterations in patients, with a subsequent impairment in affective and cognitive tasks requiring emotional or stress regulation and attention shifting [59,60]. Finally, the hippocampus, which is related to memory function, has also been shown to be impaired in depression [61], while the thalamus, which acts as a relay of information between cortical and subcortical areas and plays an important role in cognitive functions, has also been shown to be affected in depressed individuals [62].

Another finding that supports the monoamine theory relates to changes found in their key enzyme (i.e., the MAO); PET analysis has showed that depressive patients have an increase in MAO uptake in the dorsolateral prefrontal cortex, ventrolateral prefrontal cortex, thalamus, and orbitofrontal cortex [42], which could translate as an increase in monoamine degradation [63], lowering even further the metabolic rates and availability of these neurotransmitters. As neurotransmitters are necessary for normal neurological functioning, monoamine alterations as evidenced by PET studies could further consolidate the current understanding of dysfunction in these key areas and serve as a fruitful avenue for future research endeavors. Furthermore, findings in PET studies with regard to monoamine pathways vary depending on the treatment being assessed. These include not only pharmacologic treatments but also other therapies, such as transcranial magnetic stimulation and electroconvulsive therapy. Concretely, patients using SSRI showed an increase in serotonin uptake in the orbitofrontal cortex, amygdala, hippocampus, and cingulate cortex, among other structures [64], and a decrease in serotonin uptake in the basal ganglia and limbic cortex, among other structures [65]. The same was seen with patients subjected to electroconvulsive therapy, with a decrease in serotonin uptake after a session in occipital, parietal, and cingulate cortices [66]. Norepinephrine binding showed dose-dependent alterations, with an increase in norepinephrine binding in patients treated with venlafaxine or nortriptyline [39]. The use of SSRIs and benzodiazepines showed an increase in dopamine binding, which was not evidenced in patients that did not respond to treatment [35]. This finding is an important hallmark of this study, as one of the key objectives of psychiatric research is to find biomarkers that could potentially diagnose and/or predict the response to treatment [59,67].

Another great pillar on the current understanding of pathogenesis in various psychiatric disorders, including depression, is neuroinflammation, which intercedes in various metabolic processes [68]. Notably, depression is associated with an alteration of the kynurenine pathway, with the subsequent liberation of excitotoxic metabolites and the alteration of neurotransmission, blood–brain barrier dysfunction, and activation of microglia in close relation to alterations in cerebral blood flow [68]. Certainly, the relation between brain blood flow alterations and neuroinflammations has been studied in other neuropsychiatric diseases [69], and it comes to reason that the same relationship could also factor in depression. This framework falls in line with the findings of the included studies, where alterations were found in microglial activity, brain–blood barrier function, and cerebral blood flow with the use of PET. Concretely, studies postulate that in depressed patients, an increase in inflammation markers was associated with higher suicidal ideation [48]. Likewise, a poorer CBF was shown to have a relation with higher depressive scores on validated scales [50]. Furthermore, patients who responded favorably to diverse non-pharmacologic therapies (TMS and pain tolerance stimulation) showed an increase in cerebral blood flow in various regions of interest [70,71]. Additionally, increased microglial activity and blood–brain barrier glycoprotein disfunction was evidenced in depressed subjects with PET studies [55] (P glycoprotein and microglial activity). These empirical findings support the multifactorial pathogenesis of depression which involves neuroinflammation, blood flow alteration, and microglial disfunction, as stated above [59,67,68].

Glucose was also found to be importantly affected in depressed patients. While the literature shows conflicting findings in some regions and treatments, the mainstay is that patients with depression show a decrease in FDG uptake in various ROIs, including the cingulate and prefrontal cortices, among others [72]. Glucose abnormalities have been shown to play a key part in depression, in various age groups [72]. Furthermore, these alterations are hypothesized to relate to dysfunctions in the hypothalamic–pituitary–adrenal axis, which also plays a key role in neuroinflammation and the kynurenine pathway [68,73]. These interweaved dysregulations play a role not only in depression but also in other neuropsychiatric conditions such as bipolar disorder and Alzheimer’s disease [73,74].

Discrepancies across PET studies likely reflect a combination of biological and methodological factors rather than true inconsistencies in depressive pathophysiology. Differences in tracer specificity, outcome metrics, and analytic pipelines limit direct comparability across studies. In addition, clinical heterogeneity, including medication status at scanning, treatment resistance, age group, diagnostic subtype, and therapeutic modality, substantially influences PET signals. For example, baseline metabolic or neurochemical patterns that predict response in treatment resistant depression may differ from those observed in first-episode or late-life cohorts. Similarly, neuromodulatory interventions often produce regionally focal changes, whereas pharmacological treatments may yield more diffuse or delayed effects. These factors underscore the need to interpret PET findings within their specific clinical and methodological context rather than as uniform biomarkers; it also showcases the need to pursue standardization in future endeavors if the scientific community wishes to use nuclear imaging as a potential biomarker.

### 4.1. Integrative Interpretation of Metabolic, Neurochemical, Inflammatory, and Perfusion PET Findings in Depression

Although PET studies in depression have traditionally been interpreted within isolated biological domains, the available evidence points towards a model in which energetic, neurochemical, inflammatory, and perfusion abnormalities converge within shared cortico-limbic networks. Across glucose alterations, monoaminergic transmission, neuroinflammation, and cerebral blood flow studies, recurrent involvement of the anterior cingulate cortex, prefrontal cortex, limbic structures, basal ganglia, and thalamus emerges as a unifying anatomical substrate of depressive pathology and treatment response [1,2,5,10,59,60]. These regions constitute interconnected circuits responsible for emotional regulation, cognitive control, reward processing, and stress integration, suggesting that PET-detected abnormalities reflect network-level dysfunction rather than isolated molecular defects. Furthermore, alterations in cerebral glucose provide a foundational energetic context for interpreting other PET findings; reduced or dysregulated FDG uptake in prefrontal and cingulate regions has been associated with depressive symptom severity, suicidal ideation, and impaired cognitive performance [5,10,11,14,15]. Importantly, treatment-induced metabolic shifts in these same regions have been observed following pharmacological, psychotherapeutic, and neuromodulatory interventions, indicating that energetic metabolism is dynamically linked to clinical state rather than representing a static trait marker [7,8,9,16,19]. These changes likely reflect downstream consequences of altered synaptic activity driven by monoaminergic and inflammatory processes.

Certainly, monoaminergic PET findings align with this interpretation. Serotonergic, dopaminergic, and noradrenergic uptake alterations are repeatedly detected within regions that also demonstrate glucose and perfusion abnormalities, particularly the anterior cingulate cortex and prefrontal cortex [1,2,21,23,32,37]. Reduced serotonin receptor availability or transporter binding, altered dopamine transporter density, and changes in norepinephrine transporter occupancy suggest impaired neuromodulatory regulation of these networks [23,28,30,32,37,38]. Elevated monoamine oxidase A and B binding also suggests a state of enhanced monoamine degradation, potentially amplifying neurotransmitter deficits and contributing to reduced synaptic efficiency and metabolic demand [40,41,42]. The observation that successful antidepressant treatments modulate monoamine-related PET measures in parallel with glucose and perfusion changes reinforces the concept of interdependent biological systems rather than independent pathways [6,7,16,35,43].

Neuroinflammatory PET findings provide an additional layer of integration. Increased translocator protein binding in the anterior cingulate cortex, prefrontal cortex, and insula has been associated with longer duration of untreated depression and greater symptom burden [48,54]. Neuroinflammation is known to influence monoamine synthesis and metabolism through activation of the kynurenine pathway and modulation of monoamine oxidase activity, while also affecting cerebral blood flow and glucose utilization [61,68]. Within this framework, inflammatory PET signals may represent a mechanistic bridge linking neurotransmitter dysregulation and energetic impairment within vulnerable neural circuits. Cerebral blood flow abnormalities further complement this model; PET studies demonstrate altered perfusion in prefrontal, cingulate, temporal, and limbic regions in depressed patients, with perfusion changes correlating with symptom severity and treatment response [3,17,18,50,51,52,53]. Because cerebral blood flow is tightly coupled to metabolic demand and synaptic activity, perfusion abnormalities likely reflect the combined influence of neurotransmitter dysfunction, neuroinflammation, and altered neuronal energetics rather than an isolated vascular phenomenon [69,70,71].

Taken together, PET evidence suggests a network-based pathophysiological model of depression in which disturbances in glucose metabolism, monoaminergic transmission, neuroinflammation, and cerebral blood flow interact within shared cortico-limbic circuits. Differences between treatment responders and non-responders across multiple PET domains suggest that effective therapies restore network-level balance rather than targeting a single molecular abnormality [2,5,8,26,67]; however, more studies looking into these two groups are necessary to further elucidate their metabolic differences as most studies have not focused in properly dividing these groups and thoroughly studying their metabolic PET alterations. We believe that framing PET findings within this integrative context improves interpretability and underscores the potential of multimodal metabolic profiling to inform treatment stratification and mechanistic understanding of depressive disorders if further studied and standardized.

### 4.2. Limitations

Our study has various limitations. First, the methodology of this study, being a literature review, was not as robust as a systematic review; however, we have tried to mitigate this by providing a Section 2 and transparently explaining our selection process. Second, the studies herein synthetized have a relatively small sample of subjects, given the complexity and high economic cost of the technique (PET). Third, most studies do not account for potentially biasing comorbid psychiatric conditions and duration of treatment. Fourth, the heterogeneity of the clinical evaluation of depressive symptoms in the different included students could not be controlled as each center and professional can perform different evaluations, and standardization is urgently needed in the psychology/psychiatric fields in order to produce reliable and reproducible research; while most studies use different scales for assessment, most consider DSM symptoms, which may overlook other, non-DSM specified indicators that are also central to depression [75]. Fifth, several limitations inherent to PET imaging warrant consideration when interpreting the present findings. PET tracers provide indirect measures of biological processes and do not permit direct observation of underlying molecular cascades. For example, TSPO ligands are commonly used as markers of neuroinflammation but primarily reflect translocator protein expression, which is not specific to activated microglia and may also be expressed by astrocytes and endothelial cells. Similarly, PET cannot distinguish pro-inflammatory from anti-inflammatory microglial states. Measures of cerebral blood flow capture neurovascular coupling rather than neuronal metabolism per se, and blood–brain barrier function can only be inferred indirectly using specialized tracers rather than assessed directly. Importantly, while monoaminergic and monoamine oxidase PET studies inform on receptor availability, transporter binding, or enzyme density, they do not directly measure neurotransmitter release, synaptic concentrations, or downstream pathways such as kynurenine metabolism. Consequently, PET findings should be interpreted as biomarkers of system-level alterations rather than definitive evidence of causal mechanisms. Finally, the breadth of this review necessarily limits the depth of mechanistic interpretation for each individual pathway; however, we considered this trade-off acceptable given our objective of providing a unifying overview of PET-based signatures in depression.

## 5. Conclusions

PET studies in depressive disorders consistently report alterations across energetic, neurochemical, inflammatory, and perfusion-related domains, most prominently within prefrontal, cingulate, limbic, and striatal circuits. Across FDG PET investigations, regional differences in glucose utilization are frequently observed at baseline and, in some studies, distinguish treatment responders from non-responders. Neurochemical PET studies similarly report alterations in monoaminergic systems and monoamine oxidase-related measures, although the direction and regional distribution of these findings vary according to tracer, clinical phenotype, and analytic approach. PET markers of neuroinflammation and cerebral blood flow further indicate involvement of neuroimmune and neurovascular processes, but their interpretation remains constrained by tracer specificity and methodological heterogeneity. Importantly, the extent to which PET alterations reflect baseline characteristics, treatment-associated changes, or state-dependent phenomena depends on study design. Longitudinal studies with pre- and post-treatment imaging provide evidence of treatment-related modulation in specific regions, whereas cross-sectional studies primarily support associations with clinical status or outcome rather than causal inference. Consequently, PET findings should be interpreted as biomarkers associated with depressive phenotypes and treatment response, not as definitive indicators of underlying mechanisms. Taken together, the reviewed evidence supports a network-level model of depression in which energetic, monoaminergic, inflammatory, and perfusion abnormalities interact within shared cortico-limbic circuits rather than representing isolated pathophysiological processes. Overall, PET imaging offers valuable insight into distributed brain alterations associated with depression, but variability in tracers, metrics, clinical populations, and study designs limits direct comparability across studies. Future research using harmonized acquisition protocols, clearer clinical stratification, and longitudinal designs will be essential to refine the role of PET-derived measures in the study and potential clinical application of depressive disorders.

## Figures and Tables

**Table 1:** Summary of the direction of PET-derived alterations reported across studies. Findings reflect baseline differences, treatment related changes, or responder versus non-responder contrasts as specified in the original articles. Directional arrows represent relative increases or decreases in the PET metric reported by each study, including FDG-based metrics, receptor or transporter binding potential, occupancy, distribution volume, or relative perfusion indices, and should not be interpreted as interchangeable quantitative measures (i.e., the arrows reflect relative changes in the reported PET metrics and should not be interpreted as direct measures of neurotransmitter concentrations or absolute metabolic rates).

Treatment Modality	Glucose Alterations	Serotonin Alterations	Dopamine Alterations	Norepinephrine Alterations	MAO Alterations
**Transcranial magnetic stimulation (TMS)**	↑ PFC, OFC, subgenual and anterior cingulate, insula, temporal lobe, parahippocampus, amygdala; ↓ PFC, OFC, left dlPFC, temporal lobes, left ACC, right medial temporal lobe	↓ Right hippocampus, right dlPFC	Not reported	Not reported	Not reported
**Deep brain stimulation (DBS)**	↑ PFC	Not reported	Not reported	Not reported	Not reported
**Vagal nerve stimulation + SSRIs**	↓ Left medial frontal gyrus, subcentral area, temporal gyri, caudate head, limbic system, brainstem	Not reported	Not reported	Not reported	Not reported
**SSRIs (general)**	↓ Midbrain, parahippocampal gyrus, putamen, globus pallidus, ventral limbic structures, thalamus, inferior frontal gyrus, cerebellum, ACC, medial and dlPFC	↑ Binding potential in dorsal, subgenual and pre subgenual ACC and medial orbital cortex; ↓ global serotonin binding availability	↑ D2 receptor binding in ACC, frontal regions and striatum in responders; ↓ same regions in non-responders	Not reported	Not reported
**Venlafaxine**	↑ Pregenual to subgenual cingulate region in non-responders; ↓ right posterior temporal gyrus and bilateral superior temporal gyri	Not reported	Not reported	↑ NET occupancy up to 150 mg; ↓ occupancy at higher doses	Not reported
**Paroxetine**	↑ dlPFC, vlPFC, parietal cortex, dorsal ACC; ↓ subgenual cingulate, anterior and posterior insula, hippocampus, parahippocampus, caudate, putamen, antero-inferior temporal lobe	↓ Medial frontal gyrus, lateral OFC, temporal regions, basal ganglia, brainstem, rostral ACC, vlPFC	Not reported	Not reported	Not reported
**Sertraline**	↑ Left lateral orbital cortex, amygdala, PCC; ↓ right inferior frontal gyrus, cerebellum, ACC, medial, and dlPFC	Not reported	Not reported	Not reported	Not reported
**Citalopram/Escitalopram**	↑ Putamen, occipital cortex, cerebellum; ↓ middle frontal cortex, inferior parietal lobule	↑ Amygdala–anterior hippocampus complex, habenula, putamen, OFC, anterior and subgenual ACC, insula, midbrain, hippocampus, globus pallidum	Not reported	Not reported	Not reported
**Clonidine**	↓ Cerebellum, thalamus	Not reported	Not reported	Not reported	Not reported
**Nortriptyline**	Not reported	Not reported	Not reported	↑ NET occupancy	Not reported
**Benzodiazepines/lithium (bipolar depression)**	↓ Lateral frontal, lateral and medial temporal cortices, basal ganglia, thalamus	Not reported	Not reported	Not reported	Not reported
**Cognitive behavioral therapy (CBT)**	↑ Occipital temporal cortex, insula, temporal lobe; ↓ OFC, medial PFC	Not reported	Not reported	Not reported	Not reported
**Psychodynamic psychotherapy**	↓ Insula	Not reported	Not reported	Not reported	Not reported
**Moclobemide (MAO inhibitor)**	Not reported	Not reported	Not reported	Not reported	↓ MAO density in all brain regions
**Electroconvulsive therapy (ECT)**	Not reported	Not reported	Not reported	Not reported	No change in MAO distribution volume

## Data Availability

No new data were created or analyzed in this study. Data sharing is not applicable to this article.
